# Enhanced Photo-Fenton Removal of Oxytetracycline Hydrochloride via BP/Bi_2_MoO_6_ Z-Scheme Heterojunction Photocatalyst

**DOI:** 10.3390/ijms26167751

**Published:** 2025-08-11

**Authors:** Jian Feng, Xiaohui Li, Xia Ran, Li Wang, Bo Xiao, Rong Li, Guangwei Feng

**Affiliations:** School of Basic Medical Sciences/School of Medical Humanities, Guizhou Medical University, Guiyang 550025, China; jfeng@gmc.edu.cn (J.F.); lxh530518573@hotmail.com (X.L.); ranxia@gmc.edu.cn (X.R.); mona19851228@126.com (L.W.); xiaobogzmu@163.com (B.X.)

**Keywords:** BP/Bi_2_MoO_6_, Z-scheme heterojunction, photo-Fenton degradation, oxytetracycline

## Abstract

Fenton oxidation technology utilizing hydrogen peroxide is recognized as an effective method for producing reactive oxygen species (ROS) to facilitate the degradation of antibiotics. However, the requirement for strongly acidic conditions during this process significantly restricts its broader applicability. In this study, we synthesized black phosphorus (BP) nanosheets by exposing the {010} crystal planes and then constructed a 0D/2D BP/Bi_2_MoO_6_ (PBMO) heterojunction to function as a Fenton catalyst. The PBMO-75 heterojunction exhibited a remarkable increase in photo-Fenton catalytic activity towards oxytetracycline (OTC) under neutral conditions, achieving catalytic efficiencies that were 20 and 8 times greater than those of BP and Bi_2_MoO_6_ (BMO), respectively. This can be attributed to its strong absorption of visible light, the establishment of an internal electric field (IEF) at the interface, and the implementation of a Z-scheme catalytic mechanism. Additionally, the photo-Fenton system was further improved in OTC degradation through the continuous conversion of Mo^6+^/Mo^5+^ under visible light irradiation in conjunction with H_2_O_2_. Based on ERS, XPS, and active species trapping experiments, we propose a Z-scheme charge transfer mechanism for PBMO. This research offers compelling evidence that 0D/2D Z-scheme heterojunctions are promising candidates for the photo-Fenton treatment of antibiotic contaminants.

## 1. Introduction

With the rapid population growth and industrialization in modern society, water pollution has intensified significantly. Antibiotics represent a prevalent class of emerging pollutants in the environment [1]. In recent years, their “pseudo-persistence” and capacity to induce antibiotic resistance genes and bacteria within environment have posed substantial threats to human health and ecological safety [2,3]. Consequently, water pollution resulting from the excessive use of antibiotics has emerged as a major environmental challenge confronting humanity. The development of simple, efficient, and cost-effective water purification technologies has become a critical priority for sustainable human development. Traditional technologies for the removal of antibiotic contaminants include adsorption [4], membrane filtration [5], and biodegradation [6], while more recent approaches such as electrocoagulation are also gaining attention [7]. These technologies have several drawbacks, including low efficiency, high expenses, operational complexity, and inevitable secondary pollution. Advanced oxidation methods, such as the Fenton reaction [8,9] and photocatalysis [10,11,12], have shown promise as effective approaches for addressing antibiotic pollution. However, the traditional Fenton reaction faces challenges, including the requirement for a strongly acidic environment and the tendency of iron ions to precipitate [13]. Recently, the development of heterogeneous photo-Fenton systems has attracted widespread attention, as these systems address the limitations of traditional Fenton oxidation and synergistically facilitate the removal of refractory pollutants under visible light irradiation. Nevertheless, as a critical factor in the practical application of the photo-Fenton method, the development of semiconductor catalysts with high stability and efficiency remains essential [14,15].

Over recent decades, a variety of semiconductor catalysts have been developed for the photo-Fenton oxidation of antibiotics, including graphitic carbon nitride [16,17], metal sulfides [18,19], perovskite oxides [20,21], and metal–organic frameworks [22,23], among others. Black phosphorus (BP) is characterized by a graphite-like layered structure, wherein bulk BP consists of stacked layers held together by van der Waals forces. Thin BP nanosheets can be obtained via exfoliation techniques. Notably, the band gap of BP is thickness-dependent, ranging from 0.3 eV in bulk form to 2.0 eV in monolayer form [24]. Additionally, BP exhibits strong optical absorption spanning the ultraviolet to near-infrared (NIR) regions, coupled with high carrier mobility. These properties have rendered BP a subject of extensive investigation as a promising photocatalyst over the past decade [25,26]. However, BP also presents certain limitations that hinder its widespread application in photocatalysis. Specifically, thin-layered BP nanosheets are highly sensitive to light, water, and oxygen [24], which compromises their chemical stability during photocatalytic processes. Furthermore, the negative valence band position of BP results in limited oxidative capability. Pristine BP is also prone to rapid recombination of photogenerated charge carriers, further restricting its practical utility. Therefore, strategies aimed at suppressing charge carrier recombination, enhancing oxidative capacity, and improving the stability of BP nanosheets are critical for their effective deployment in photocatalytic applications.

Bismuth molybdate (Bi_2_MoO_6_, BMO) is a semiconductor with a tunable band gap of 2.6–2.8 eV, indicating that BMO exhibits a visible light response. BMO has been widely utilized in photocatalytic organic pollutant degradation, water splitting, and sterilization [27]. BMO exhibits low toxicity, high chemical stability, ease of preparation, and photostability [28]. Especially, BMO has more positive VB potential than that of BP, which probably implies that BMO is a suitable semiconductor material for constructing a heterojunction with BP. DFT calculation results indicate that the VB of BMO is composed of O 2p orbitals, while the conduction band (CB) is constituted by Bi 6p and Mo 4d orbitals [29]. Therefore, the photo-absorption and electron transfer properties of BMO are sensitive to the ratio of Bi, O, and Mo [30]. Although a variety of semiconductors such as g-C_3_N_4_ [31], MoS_2_ [32], TiO_2_ [33], CoP [34], Bi_2_WO_6_ [35], etc. have been used to form heterojunctions with BP, there has been limited research on BP/BMO heterojunctions. In this study, we fabricated BP/BMO heterojunctions using a facile method at room temperature. The visible light-driven photo-Fenton oxidation activity was assessed by degrading oxytetracycline (OTC).

## 2. Results

A series of 0D/2D PBMO heterojunctions was synthesized through a multi-step procedure. Initially, bulk BP was subjected to milling in anhydrous ethanol for a duration of 6 h. Subsequently, larger BP particles were removed via centrifugation, isolating smaller BP particles that were then exfoliated by sonication in an ice bath with N-methyl-2-pyrrolidone (NMP) for 8 h to yield BP nanosheets. Thereafter, 100 mg of bismuth molybdate (BMO) was introduced into varying volumes (30, 60, 75, 90, and 120 mL) of the BP NMP dispersion, followed by sonication for 3 h and continuous stirring overnight. The resulting mixtures were centrifuged, washed thrice with ethanol, and dried naturally in the dark for 12 h. The heterojunction samples obtained were designated as PBMO-30, PBMO-60, PBMO-75, PBMO-90, and PBMO-120, corresponding to the volume of BP solution used.

**Figure 1:**
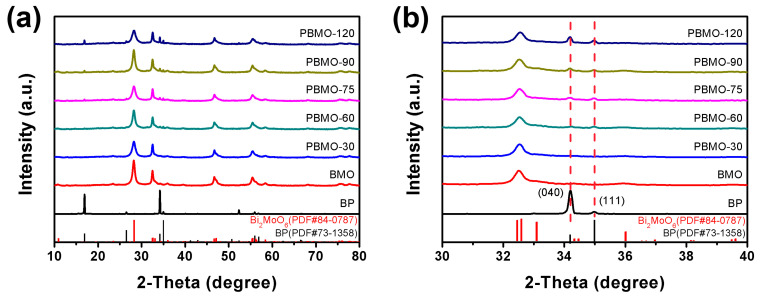
(**a**) XRD patterns and (**b**) a partially enlarged plot of the BP, BMO, and PBMO heterojunctions.

Phase identification of BP, BMO, and the PBMO heterojunctions was conducted using X-ray diffraction (XRD). The orthorhombic crystal structures of pristine BP (PDF#73-1358) [36,37] and BMO (PDF#84-0787) [38,39] were confirmed, demonstrating the successful synthesis of pure-phase materials (Figure 1a). The PBMO heterojunctions exhibited diffraction peaks characteristic of both BP and BMO. Notably, peak broadening observed in the diffraction patterns of BMO and PBMO heterojunctions suggests the presence of nanoscale crystallite dimensions. Additionally, the intensity of BP diffraction peaks at 34.2° and 35.0°, corresponding to the (040) and (111) crystallographic planes, respectively, increased proportionally with the BP content in the heterojunctions (Figure 1b). These findings collectively confirm the coexistence and successful integration of BP and BMO phases within the PBMO heterojunctions.

**Figure 2:**
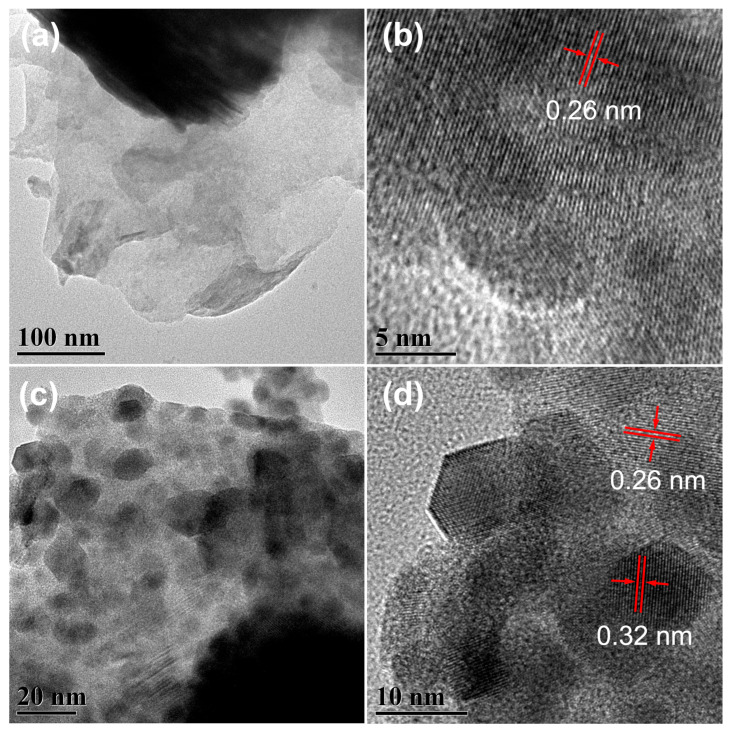
(**a**) TEM and (**b**) HRTEM images of BP; (**c**) TEM and (**d**) HRTEM images of the PBMO-75 heterojunction.

The thin-layered structure of BP nanosheets is evident in the transmission electron microscopy (TEM) image (Figure 2a). The observed lattice fringes exhibit a spacing of 0.26 nm, corresponding to the (040) crystal plane of BP (Figure 2b). This observation confirms the exposure of the {010} crystal planes, which is consistent with the XRD results presented in Figure 1b. The thin-layered structure of BP is also evident in the PBMO-75 heterojunction (Figure 2c), where BMO nanoparticles were deposited onto the surface of BP, with particle sizes ranging from 10 to 20 nm. This finding provides direct evidence for the successful construction of PBMO heterojunctions. The high-resolution transmission electron microscopy (HRTEM) image of PBMO-75 reveals a distinct close contact between BP and BMO, with lattice fringes displaying a spacing of 0.32 nm, corresponding to the (131) crystal plane of BMO. The homogeneous structure of the PBMO-75 heterojunction was further confirmed using scanning electron microscopy (SEM) images (Figure S1). The layered structure of BP and the surface adhesion of BMO were clearly observed. Energy-dispersive spectroscopy (EDS) was utilized to determine the chemical composition of the PBMO-75 heterojunction. The analysis revealed characteristic peaks corresponding to the elements P, Bi, Mo, and O (Figure S2). The distribution of P, Bi, Mo, and O in PBMO-75 was evaluated through elemental mapping. The uniformity observed in the elemental mapping images indicates that P, Bi, Mo, and O are evenly distributed throughout PBMO-75 (Figure S3). These findings confirm the formation of a two-dimensional (2D) heterojunction between BP and BMO.

**Figure 3:**
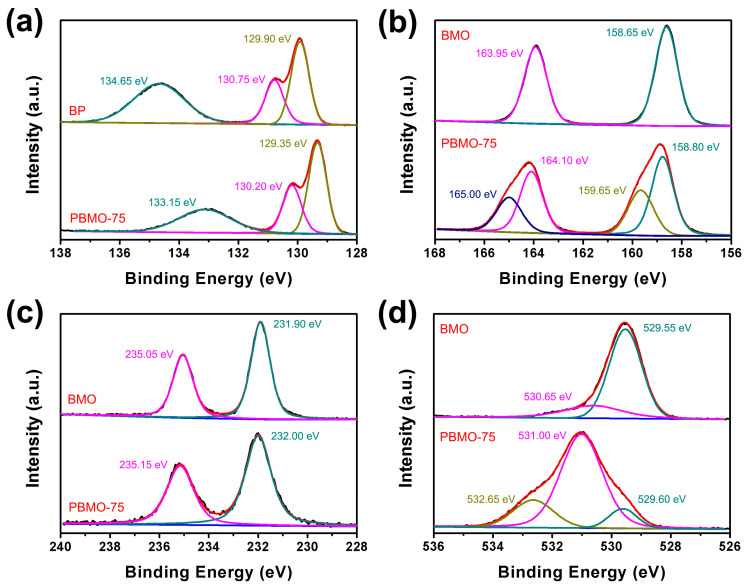
High-resolution XPS spectra of BP, BMO, and the PBMO-75 heterojunction: (**a**) P 2p, (**b**) Bi 4f, (**c**) Mo 3d, and (**d**) O 1s.

The chemical composition and valence states of the elements present in the BP, BMO, and PBMO-75 heterojunction were analyzed using X-ray photoelectron spectroscopy (XPS). All binding energies were calibrated against the C 1s peak at 284.60 eV. As depicted in the survey spectra (Figure S4), the presence of P, Bi, Mo, and O was confirmed in the PBMO-75 heterojunction, indicating that this sample comprises both BP and BMO. The analysis further reveals that BP contains P and O, with the latter resulting from the surface oxidation of BP nanosheets. High-resolution spectra for P 2p, Bi 4f, Mo 3d, and O 1s were analyzed. The characteristic peaks for P 2p_3/2_ and P 2p_1/2_ of BP were identified at 129.90 eV and 130.75 eV, respectively (Figure 3a), while a peak at 134.65 eV was attributed to oxidized phosphorus (P_x_O_y_) [40]. In the PBMO-75 sample, these peaks exhibited a notable shift to 129.35 eV, 130.20 eV, and 133.15 eV compared to pristine BP. The Bi 4f spectra for BMO and PBMO-75 are illustrated in Figure 3b, where two distinct peaks for Bi 4f_7/2_ and Bi 4f_5/2_ of BMO were observed at 158.65 eV and 163.95 eV [41], respectively, indicating the presence of Bi^3+^. In the PBMO-75 sample, these Bi 4f peaks shifted to 158.80 eV and 164.10 eV. Additionally, Figure 3c demonstrated that the binding energies at 235.05 eV and 231.90 eV correspond to the characteristic peaks of Mo 3d_3/2_ and Mo 3d_5/2_ [42], respectively, suggesting the presence of Mo^5+^ and Mo^6+^ [39]. These peaks shifted to 235.15 eV and 232.00 eV in the PBMO-75 heterojunction.

The O 1s spectral peaks for BMO, located at 529.55 eV and 530.65 eV, can be attributed to lattice oxygen and oxygen vacancies, respectively (Figure 3d) [39,43,44]. In comparison to pristine BMO, the PBMO-75 heterojunction exhibited an increase in oxygen vacancies, indicating that the formation of the PBMO heterojunction contributed to the enhancement of oxygen vacancies within BMO. This phenomenon may be attributed to the stronger coordination of Bi and Mo on the surface of BMO with BP, which, in turn, reduces the surface oxygen content. The binding energies of Bi 4f, Mo 3d, and O 1s in the PBMO-75 heterojunction were found to be elevated relative to those in pristine BMO. Conversely, the binding energies of P 2p in the PBMO-75 heterojunction were lower than those observed in pristine BP. These results suggest a significant interaction between BP and BMO within the heterojunction, thereby confirming the successful formation of the PBMO heterojunction. Additionally, the variations in the binding energies of P 2p, Bi 4f, Mo 3d, and O 1s imply that electrons from the CB of BMO were drawn towards the VB of BP due to their close contact [45,46].

**Figure 4:**
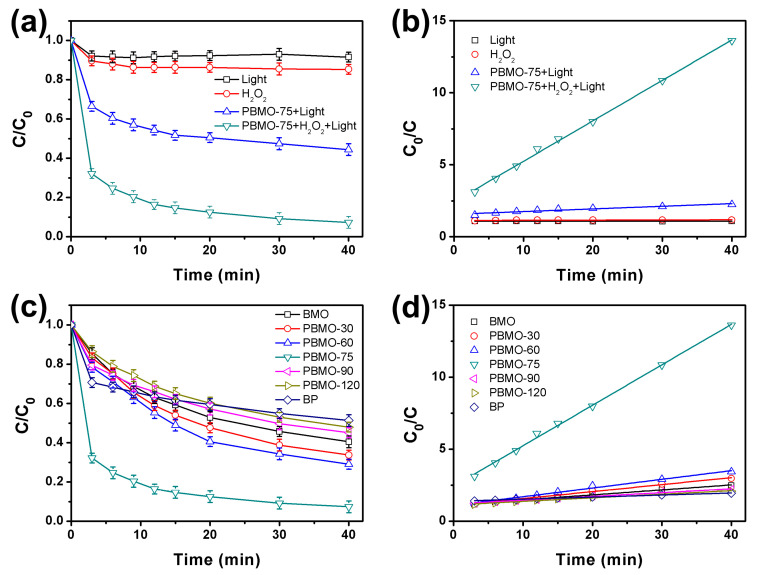
(**a**) Control experiments of the photo-Fenton degradation reaction; (**b**) the associated apparent rate constants for the degradation of OTC utilizing the PBMO-75 heterojunction; (**c**) the photo-Fenton degradation process of OTC under 40 W LED irradiation, with an OTC concentration of 20.0 mg/L, a volume of 200 mL, an initial pH of 7.0, and a H_2_O_2_ concentration of 50.0 mM; and (**d**) the corresponding apparent rate constants.

The performance of visible light-assisted photo-Fenton degradation using BP, BMO, and PBMO-75 heterojunctions was evaluated with OTC as the model pollutant. Initially, control experiments were conducted to assess the effects of light, hydrogen peroxide, and the catalysts on the efficiency of OTC degradation. As illustrated in Figure 4a, only a minimal degradation of OTC was observed when light or H_2_O_2_ was applied independently. However, the presence of the PBMO-75 heterojunction, in conjunction with illumination, facilitated a photocatalytic degradation process. Furthermore, the addition of H_2_O_2_ transformed the process into a photo-Fenton oxidation mechanism. The degradation efficiencies of OTC were recorded at 55.6% for photocatalysis and 92.9% for photo-Fenton oxidation, indicating that PBMO-75 significantly enhanced the degradation of OTC. The kinetics of OTC degradation are presented in Figure 4b, demonstrating that both photocatalytic and photo-Fenton degradation processes adhered to a pseudo-second-order kinetic model, expressed as c_0_/c = k_app_t + a [47], where t represents the degradation time (min), c_0_ denotes the initial concentration of OTC, and c indicates the concentration of OTC at time t. The apparent rate constant (k_app_) was determined to be 0.0078 (mg L^−1^)^−1^ h^−1^ for light alone, 0.072 (mg L^−1^)^−1^ h^−1^ for H_2_O_2_ alone, 1.08 (mg L^−1^)^−1^ h^−1^ for photocatalytic degradation, and 16.80 (mg L^−1^)^−1^ h^−1^ for photo-Fenton degradation. Notably, the k_app_ for the photo-Fenton oxidation was approximately 15.6 times greater than that for the photocatalytic degradation reaction.

Figure 4c illustrates the efficiency of the photo-Fenton reaction for the degradation of OTC over BP, BMO, and PBMO heterojunctions. The percentages of OTC adsorption at adsorption–desorption equilibrium were approximately 29.2%, 15.0%, 17.0%, 21.0%, 20.5%, 18.0%, and 13.7% for BP, BMO, PBMO-30, PBMO-60, PBMO-75, PBMO-90, and PBMO-120, respectively. The Brunauer–Emmett–Teller (BET) surface areas of BP, BMO, and PBMO-75 were determined using N_2_ adsorption–desorption measurements. The results, as illustrated in Figure S5, indicate the mesoporous nature of these samples [48]. The BET surface areas of BP, BMO, and PBMO-75 were measured as 59.02, 36.47, and 44.77 m^2^ g^−1^, respectively, corroborating that PBMO-75 possesses a higher adsorption capacity than BMO. The corresponding degradation efficiencies were 48.7%, 59.5%, 66.3%, 70.9%, 92.9%, 54.9%, and 52.1%. The significant enhancement in degradation efficiency for the PBMO-75 heterojunction indicates a marked improvement in charge separation efficiency within the heterojunction. Overall, the photocatalytic performance of PBMO-75 is comparable to—and, in certain cases, surpasses—that of other established photocatalytic systems (Table S1). This improvement may be attributed to the interfacial interactions between BP and BMO, which facilitate the formation of an internal electric field, thereby providing an additional driving force for the separation of photogenerated electrons and holes. Figure 4d presents the corresponding kinetic plots for OTC degradation over the BP, BMO, and PBMO heterojunctions, which conform to a pseudo-second-order kinetic model. The rate constants were determined to be 0.84 (mg L^−1^)^−1^ h^−1^ (BP), 2.10 (mg L^−1^)^−1^ h^−1^ (BMO), 2.88 (mg L^−1^)^−1^ h^−1^ (PBMO-30), 3.60 (mg L^−1^)^−1^ h^−1^ (PBMO-60), 16.80 (mg L^−1^)^−1^ h^−1^ (PBMO-75), 1.56 (mg L^−1^)^−1^ h^−1^ (PBMO-90), and 1.50 (mg L^−1^)^−1^ h^−1^ (PBMO-120), respectively. The rate constant for the photo-Fenton degradation on PBMO-75 was approximately 20 times greater than that of BP and 8 times greater than that of BMO. The Langmuir–Hinshelwood model was employed to characterize the degradation kinetics of OTC over PBMO-75, as illustrated in Figure S6 [49,50]. The surface reaction rate constant (k_r_) and adsorption equilibrium constant (K) were determined to be 20.86 mg L^−1^ h^−1^ and 6.15 L mg^−1^, respectively. These values indicate that OTC undergoes synergistic degradation on the surface of PBMO heterojunctions through a combination of adsorption and the photo-Fenton reaction.

**Figure 5:**
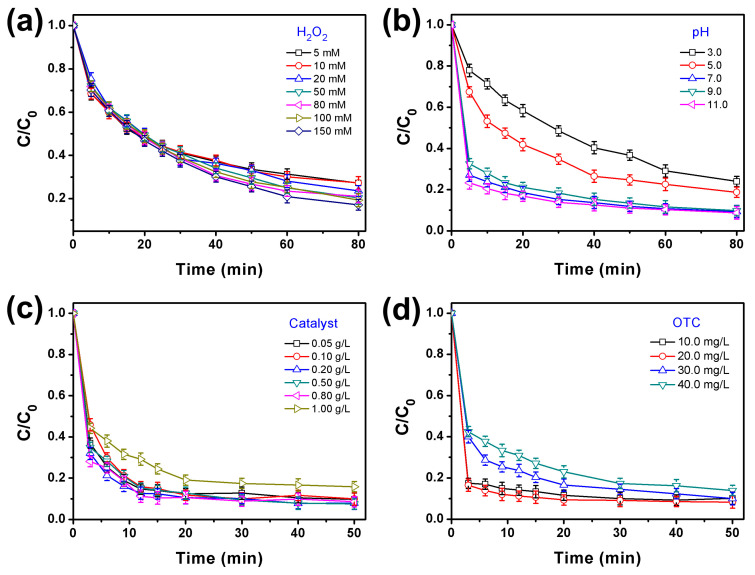
The influence of various parameters on the efficiency of visible light-assisted Fenton degradation of OTC over the PBMO-75 heterojunction includes (**a**) the concentration of H_2_O_2_ with a catalyst dosage of 0.5 g/L, an OTC concentration of 20.0 mg/L, and a pH of 5.0; (**b**) the initial pH of the solution, maintaining H_2_O_2_ at 10.0 mM, a catalyst dosage of 0.5 g/L, and an OTC concentration of 20.0 mg/L; (**c**) the catalyst dosages while keeping H_2_O_2_ at 10.0 mM, OTC at 20.0 mg/L, and the initial pH at 5.0; and (**d**) the concentration of OTC, with H_2_O_2_ set at 10.0 mM, a catalyst dosage of 0.5 g/L, and pH at 5.0.

The influence of various parameters on the efficiency of visible light-assisted Fenton degradation of OTC over the PBMO-75 heterojunction was investigated. These parameters included the concentrations of H_2_O_2_ and OTC, the initial pH, and the dosage of the catalyst. As illustrated in Figure 5a, the degradation efficiencies of OTC were assessed at H_2_O_2_ concentrations ranging from 5 to 150 mM. The findings indicated that a concentration of 50.0 mM H_2_O_2_ yielded relatively high degradation efficiency while minimizing H_2_O_2_ consumption. This observation aligns with the existing literature, which suggests that an optimal concentration of H_2_O_2_ enhances the photo-Fenton degradation process. Conversely, excessive H_2_O_2_ in the degradation solution can result in a reactive oxygen species (ROS) scavenging effect and lead to the saturation of active sites on the catalyst surface [51]. Consequently, H_2_O_2_ concentrations exceeding 50.0 mM did not significantly enhance the degradation efficiency of OTC.

Figure 5b illustrates the degradation efficiencies of OTC at initial pH levels of 3.0, 5.0, 7.0, 9.0, and 11.0. The observed degradation efficiencies exhibited significant variations in response to changes in the initial pH of the solution, highlighting the critical role of pH in the photo-Fenton degradation process. The optimal degradation efficiency of OTC using PBMO-75 was achieved within 80 min when the initial pH of the OTC solution exceeded 7.0. This finding is particularly relevant, as one of the limitations of homogeneous Fenton reactions is the requirement for a narrow pH range around 3.0 to ensure the solubility of ferrous and ferric ions [52]. Consequently, the PBMO system presents a promising alternative for the Fenton degradation of organic pollutants. The corresponding rate constants were recorded as 2.34 (mg L^−1^)^−1^ h^−1^ (pH = 3.0), 3.12 (mg L^−1^)^−1^ h^−1^ (pH = 5.0), 5.76 (mg L^−1^)^−1^ h^−1^ (pH = 7.0), 5.74 (mg L^−1^)^−1^ h^−1^ (pH = 9.0), and 5.78 (mg L^−1^)^−1^ h^−1^ (pH = 11.0), as depicted in Figure S7. The degradation rate constants at pH levels of 7.0, 9.0, and 11.0 exhibited the highest values. OTC predominantly exists as a cationic species at pH values below 3.3, transitions to a zwitterionic form between pH 3.3 and 7.5, and assumes an anionic species at pH exceeding 7.5 [53]. The point of zero charge for PBMO-75 was identified as 6.4 (Figure S8), indicating that the surface of PBMO-75 is positively charged at pH levels below 6.4 and negatively charged above this threshold. Electrostatic repulsion between OTC and the positively charged PBMO-75 surface at pH below 6.4 results in diminished OTC adsorption, consequently leading to reduced degradation efficiency. Conversely, at pH above 6.4, the anionic form of OTC exhibits limited adsorption on the negatively charged PBMO-75 surface due to electrostatic repulsion. The observed enhancement in degradation performance under alkaline conditions relative to acidic conditions (Figure 5b) can be attributed to the distinct influence of pH on the generation and activity of reactive species [54]. Specifically, in alkaline solution, the increased concentration of OH^−^ facilitates the production of •OH, which plays a critical role in promoting OTC degradation. In contrast, acidic environments, characterized by an abundance of H^+^, lead to the formation of H_3_O_2_^+^ through reactions with H_2_O_2_, thereby diminishing the activity of essential oxidative species. Additionally, the consumption of •OH by H^+^ ions further reduces the overall degradation efficiency.

As illustrated in Figure 5c, the degradation efficiency of OTC remained relatively high when the catalyst dosage was below 1.0 g L^−1^, with the optimal degradation efficiency observed at 0.2 g L^−1^. However, at a catalyst dosage of 1.0 g L^−1^, the degradation efficiency of OTC significantly declined. This reduction can be attributed to the excessive quantity of the catalyst, which obstructed light transmission and consequently diminished the light utilization rate [55,56]. Additionally, the effect of the OTC concentration on the photo-Fenton degradation efficiency using PBMO-75 was evaluated (Figure 5d). A notable decrease in degradation efficiency was observed when the OTC concentration exceeded 20.0 mg L^−1^. This decline in efficiency may be attributed to the saturation of active sites on the catalyst’s surface, which is caused by the excess OTC and the degradation byproducts present in the solution [57].

The total organic carbon (TOC) analysis was conducted to assess the extent of OTC decomposition on PBMO-75. Figure S9 illustrates the temporal variation of TOC throughout the degradation process. The results indicate that the TOC removal efficiency reached approximately 65% within 40 min, demonstrating that PBMO-75 effectively disrupted the complex structure of OTC. The mineralization rate was lower than the photocatalytic degradation efficiency, suggesting that OTC was not fully mineralized but partially transformed into other organic compounds [58]. This outcome is likely attributable to the limited degradation time. To evaluate the feasibility of utilizing PBMO-75 in practical applications, four cycles of OTC degradation were performed, and the structural stability of PBMO-75 was analyzed using XRD, TEM, and SEM measurements. The results of the cyclic experiments are illustrated in Figure S9, which demonstrates that there were no significant changes in efficiency across the four degradation cycles. The XRD, TEM, and SEM results are presented in Figures S10, S11, and S12, respectively. The minimal differences observed between the fresh and used PBMO-75 suggest that both the chemical structure and morphology of the material exhibit a high degree of stability during OTC degradation.

**Figure 6:**
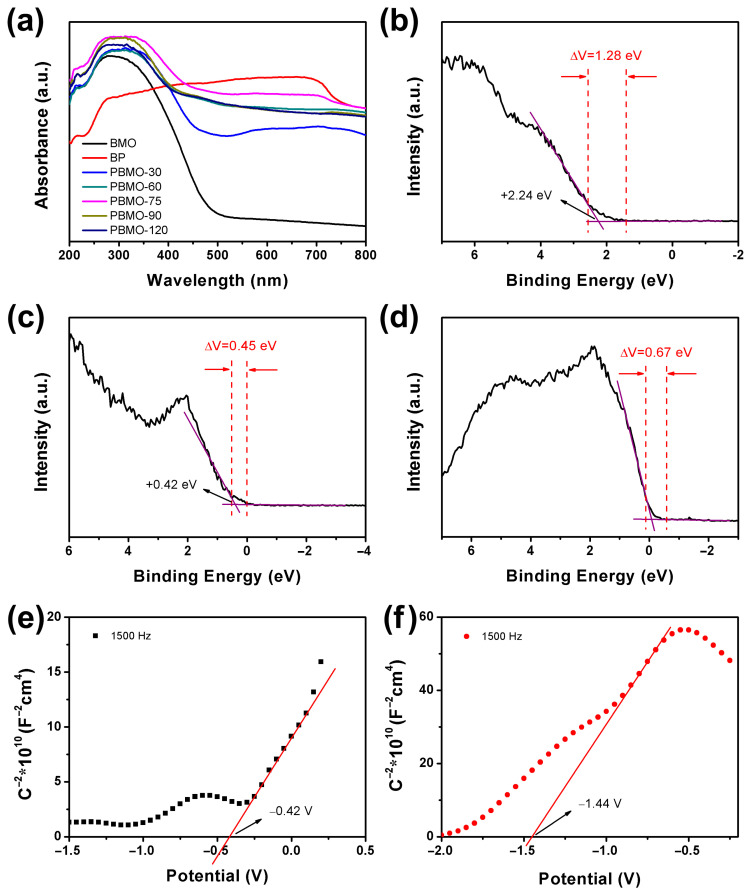
(**a**) UV–Vis DRS spectra of the as-prepared BMO, BP, and PBMO heterojunctions; VB XPS spectra for (**b**) BMO, (**c**) BP, and (**d**) PBMO-75; Mott–Schottky curves for (**e**) BMO and (**f**) BP.

The optical characteristics of the synthesized BMO, BP, and PBMO heterojunctions were evaluated utilizing UV–Vis diffuse reflection spectroscopy (DRS). As depicted in Figure 6a, BMO demonstrated the ability to absorb light across the ultraviolet to near-infrared spectrum, which is consistent with findings from previous research [39]. The formation of PBMO heterojunctions resulted in a significant enhancement of absorption within the 200–800 nm range, indicating improved light-harvesting capabilities. This enhancement suggests that PBMO heterojunctions exhibit superior light utilization efficiency. Notably, PBMO-75 displayed the highest level of light absorption, which may indicate its potential for optimal visible light-assisted Fenton degradation efficiency. In contrast, BP exhibited robust absorption across all wavelengths of light, a phenomenon attributed to its black coloration, which facilitates uniform absorption across varying wavelengths. The corresponding Tauc plots, derived from the Kubelka–Munk function, were employed to calculate the band gap energy (E_g_) values. As illustrated in Figure S13, the E_g_ of BMO was found to be 2.55 eV, indicating a strong capability for visible light responsiveness.

The configuration of energy bands in catalysts plays a crucial role in determining their catalytic efficiency. To clarify the catalytic mechanisms associated with PBMO heterojunctions, it is essential to first establish its band structure. The band potentials of PBMO were assessed through VB XPS spectra and Mott–Schottky plots. The VB values for BMO and BP were determined from the VB-XPS spectra (Figure 6b,c), yielding values of +2.24 eV and +0.42 eV, respectively. The VB potentials relative to the normal hydrogen electrode (NHE) were calculated to be +2.35 eV for BMO and +0.53 eV for BP [59]. The work functions (Φ) for BMO, BP, and PBMO-75 were assessed based on the small binding energy range scan VB-XPS spectra and can be determined using the following equation [60]:

∆V=Φ−φ

where ΔV represents the contact potential difference of the samples, while φ denotes the work function of the XPS analyzer, which was set to 4.55 eV. As illustrated in Figure 6b–d, ΔV corresponds to the interval between the two inflection points on the curve. The work functions of BMO, BP, and PBMO-75 were determined to be 5.83 eV, 5.00 eV, and 5.22 eV, respectively. The flat band potentials for BMO and BP were −0.42 eV and −1.44 eV, respectively, as obtained from the Mott–Schottky curves (Figure 6e,f). The corresponding CB potentials of BMO and BP relative to the NHE were calculated to be −0.22 eV and −1.24 eV [60]. According to the VB and CB values obtained from the XPS and Mott–Schottky results, the E_g_ of PBMO was determined to be 2.57 eV, which aligns with the band gap values measured through UV–Vis DRS, as illustrated in Figure S14. Additionally, the E_g_ of 1.77 eV for BP indicated that it exhibited a thin-layer structure, which could be attributed to the thickness-dependent band gap of BP [61].

**Figure 7:**
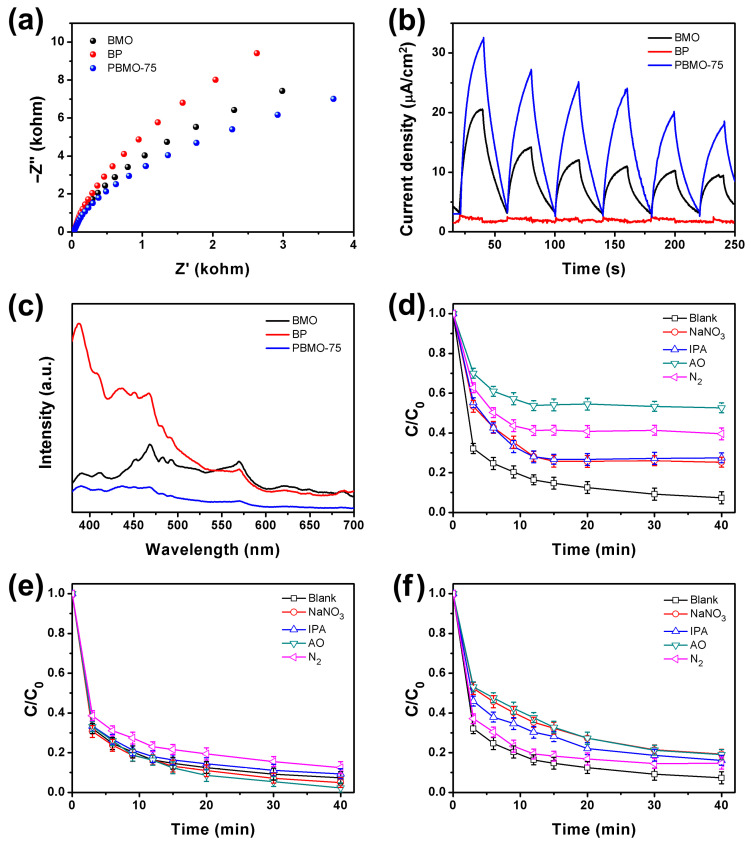
(**a**) EIS Nyquist plots; (**b**) transient photocurrent curves; (**c**) PL spectra of the synthesized BMO, BP, and PBMO-75 heterojunctions; and the influence of radical scavengers on the photo-Fenton degradation of OTC over (**d**) BP, (**e**) BMO, and (**f**) PBMO-75 heterojunctions under the irradiation of a 40 W LED.

Electrochemical assessments and photoluminescence (PL) spectroscopy were conducted to investigate the separation and migration of photogenerated charge carriers in the synthesized catalysts. The results of electrochemical impedance spectroscopy (EIS) indicated that the radius for PBMO-75 was smaller than that of the pristine BMO and BP (Figure 7a), revealing that the resistance of PBMO-75 was lower than that of BMO and BP. This finding signifies that the migration of photogenerated charges occurred more readily in PBMO-75 compared to the pristine BMO and BP. Furthermore, the photocurrent density observed for PBMO-75 was significantly higher than that of the pristine BMO and BP (Figure 7b), implying a more effective separation of photogenerated charges in PBMO-75. The PL spectra were recorded using an excitation wavelength of 360 nm (Figure 7c). Notably, the PL emission intensity for PBMO-75 was markedly lower than that of the pristine BMO and BP, indicating that the radiative transition of photogenerated electrons was effectively inhibited, thereby enhancing the efficiency of photogenerated charge separation when BMO was deposited onto BP.

**Figure 8:**
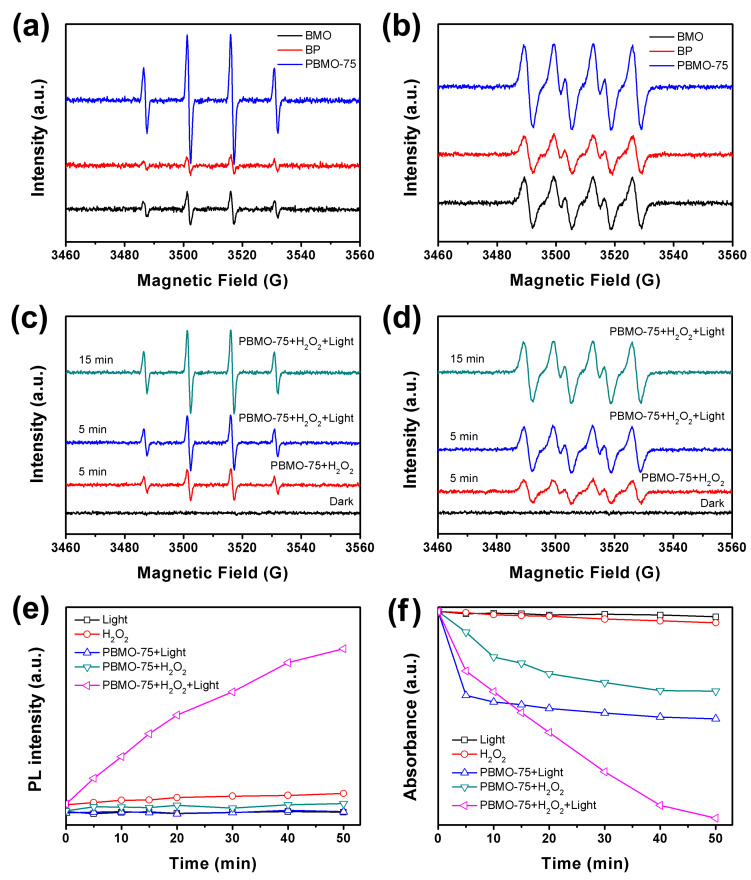
ESR spectra for (**a**) DMPO-•OH in aqueous solution and (**b**) DMPO-•O_2_^−^ in methanol, utilizing BMO, BP, and PBMO-75 within photo-Fenton systems; ESR spectra for (**c**) DMPO-•OH in aqueous solution and (**d**) DMPO-•O_2_^−^ in methanol over PBMO-75 across various systems; (**e**) PL of TAOH at 426 nm with an excitation wavelength of 312 nm; and (**f**) the absorbance of NBT at 259 nm.

Radical scavenger experiments were performed to elucidate the primary reactive species involved in the photo-Fenton degradation of OTC using BMO, BP, and PBMO-75 heterojunctions (Figure 8d–f). NaNO_3_, iso-propanol (IPA), ammonium oxalate (AO), and N_2_ were utilized as scavengers to capture e^−^, •OH, h^+^, and •O_2_^−^, respectively [20]. Figure 8d illustrates the degradation efficiencies of OTC on pristine BP in the presence of various scavengers. The introduction of NaNO_3_, IPA, AO, and N_2_ significantly diminished the OTC degradation efficiency to varying extents, indicating the involvement of e^−^, •OH, h^+^, and •O_2_^−^ in the degradation process. Notably, N_2_ exhibited a pronounced inhibitory effect on OTC degradation efficiency over BMO (Figure 8e), suggesting that •O_2_^−^ was the predominant reactive species in the photo-Fenton degradation over BMO. In Figure 8f, the addition of NaNO_3_, IPA, AO, and N_2_ led to a substantial decrease in OTC degradation efficiency, mirroring the results observed with pristine BP, thereby confirming the participation of e^−^, •OH, h^+^, and •O_2_^−^ in the photo-Fenton degradation reaction. The photogenerated holes and electrons were identified as the principal reactive species in the photo-Fenton degradation of OTC over PBMO-75, attributed to the enhanced redox capacity of the catalysts. BMO demonstrated superior OTC degradation efficiency compared to BP, likely due to its more positive valence band position relative to BP. Furthermore, PBMO-75 exhibited even higher OTC degradation efficiency than BMO, which can be attributed to the formation of a Z-scheme heterojunction that facilitates improved charge carrier separation efficiency.

The electron spin resonance (ESR) spectra of DMPO-•OH and DMPO-•O_2_^−^ were utilized to evaluate the production of •OH and •O_2_^−^ radicals within photo-Fenton systems incorporating BMO, BP, and PBMO-75. The signal intensities of DMPO-•OH for BMO and PBMO-75 were found to be greater than that of BP (Figure 8a). Notably, the DMPO-•OH signal for PBMO-75 was considerably greater than that of BMO. This enhancement can primarily be attributed to the strong interfacial interactions between BMO and BP, which facilitated improved separation of photogenerated electrons and holes within the PBMO-75 heterojunction. Similarly, the signal intensities for DMPO-•O_2_^−^ in BMO and PBMO-75 also exceeded that of BP (Figure 8b). Interestingly, BMO demonstrated a greater capacity for •O_2_^−^ generation compared to BP under identical visible light irradiation, a finding that closely parallels the results for DMPO-•OH presented in Figure 8a. This suggests that BMO could serve as a potentially effective visible light catalyst suitable for the development of heterojunctions with BP. Furthermore, PBMO-75 exhibited superior capability for •O_2_^−^ generation relative to BMO, which can be attributed to the internal electric field generated by the formation of the Z-scheme heterojunction, thereby enhancing the separation and transfer efficiency of photogenerated charges.

To further elucidate the generation mechanism of hydroxyl and superoxide radicals, ESR spectra of the PBMO-75 heterojunction were analyzed under various conditions of visible light irradiation. As illustrated in Figure 8c, the DMPO-•OH signal became detectable following the addition of H_2_O_2_ after 5 min of visible light exposure. Furthermore, the intensity of the DMPO-•OH signal exhibited a continuous increase with extended irradiation time, indicating that both visible light irradiation and the presence of H_2_O_2_ are essential for the production of •OH radicals. Comparable findings regarding the capability for •OH production are presented in Figure 8d. The intensity of the DMPO-•O_2_^−^ signal increased following the introduction of the catalyst and H_2_O_2_ under visible light irradiation. A quantitative assessment of •OH and •O_2_^−^ radicals was also conducted to support this conclusion. As depicted in Figure 8e, •OH radicals were consistently produced within the photo-Fenton system. The combination of visible light irradiation and H_2_O_2_ remarkably enhanced the production capacity of •O_2_^−^ after the addition of the catalyst (Figure 8f). The •O_2_^−^ concentration increased steadily with prolonged irradiation time when PBMO-75 and H_2_O_2_ were present. Therefore, it can be inferred that the strong interfacial interactions between BMO and BP within the PBMO heterojunctions, along with the Z-scheme junction, provide the driving force for the separation of photogenerated electrons and holes. The introduction of BMO into BP also enhanced the absorption of the visible light. Collectively, these factors facilitated the production of e^−^, •OH, h^+^, and •O_2_^−^, thereby significantly improving the efficiency of visible light-assisted photo-Fenton degradation.

**Figure 9:**
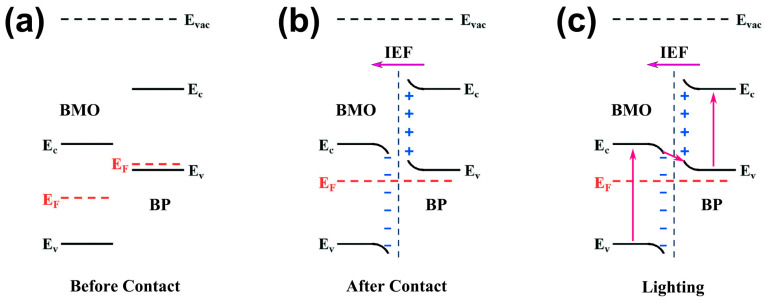
(**a**) The energy levels EC, EV, and EF of BMO and BP before contact; (**b**) the IEF and band bending at the interface of BMO and BP after contact; and (**c**) the Z-scheme transfer mechanism of photogenerated charge carriers between BMO and BP under visible light irradiation.

**Figure 10:**
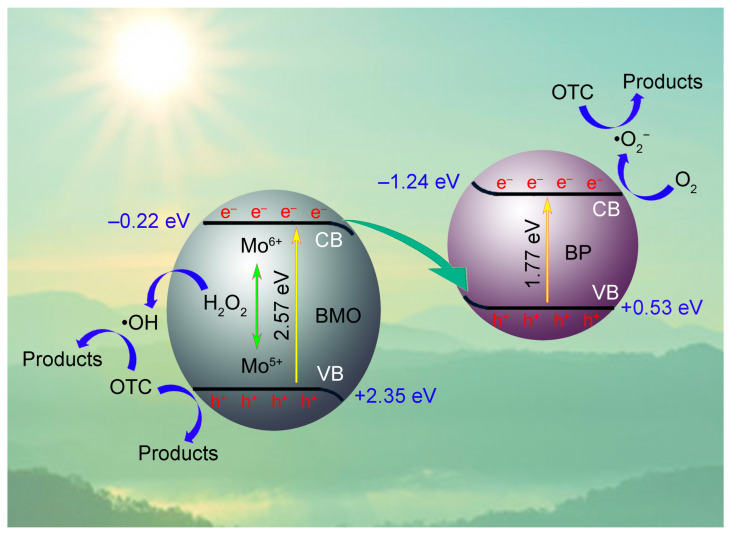
Schematic illustration of the photo-Fenton degradation of OTC over PBMO-75 under 40 W LED irradiation.

Based on the results of the Mott–Schottky curves, VB XPS spectra, and work functions, the band structure, band bending, and Z-scheme charge transfer mechanism between BMO and BP are illustrated in Figure 9. BMO exhibits a higher work function (5.83) and a lower Fermi level. In contrast, BP is characterized by a lower work function (5.00 eV) and a higher Fermi level (Figure 9a). Upon contact between BMO and BP, electrons migrate from BP to BMO to achieve equilibrium in the Fermi energy levels. Consequently, BP experiences a loss of electrons, resulting in a positive charge, while BMO acquires electrons, leading to a negative charge at the interface. This interaction establishes an internal electric field (IEF) directed from BP to BMO. Concurrently, the energy band of BP exhibits upward bending due to electron transfer, whereas the energy band of BMO bends downward as a result of electron accumulation (Figure 9b). Under visible light irradiation, photogenerated electrons in the CB of BMO are driven by the IEF to migrate towards BP, where they recombine with holes in the VB of BP (Figure 9c). Thus, a Z-scheme catalytic mechanism can be proposed. The alterations in the binding energy of P 2p, Bi 4f, and Mo 3d following the formation of the heterojunction (Figure 3), along with the Fermi levels of BMO and BP (Figure 6), indicate that electrons are transferred from BMO to BP within the PBMO heterojunction. In the absence of a Z-scheme mechanism, the holes in the VB of BP would not possess a sufficiently high potential to oxidize and generate •OH (•OH/OH^−^, 1.99 eV) [62], which contradicts the results obtained from the ERS and trapping experiments. The band bending and the IEF facilitates the transfer of electrons from the CB of BMO to the VB of BP, which enhances the separation of charge carriers, leaving holes in the VB of BMO and electrons in the CB of BP. The Z-scheme mechanism effectively utilizes the recombination of electrons in the CB of BMO and holes in the VB of BP, thereby sustaining the high redox potential of the electrons in the CB of BP and the holes in the VB of BMO. Furthermore, during the photo-Fenton reaction, the photogenerated electrons present in the VB of BMO are capable of reducing Mo^6+^ to Mo^5+^. Subsequently, Mo^5+^ can interact with H_2_O_2_ to regenerate Mo^6+^ and produce •OH, which can be utilized for the degradation of OTC (Figure 10).

## 3. Experiment

### 3.1. Chemicals and Materials

Red phosphorus, Sn, I_2_, toluene, anhydrous ethanol, N-methyl-2-pyrrolidone (NMP), Na_2_MoO_4_•2H_2_O, Bi(NO_3_)_3_•5H_2_O, cetyltrimethylammonium bromide (CTAB), and OTC were purchased from Aladdin Reagent Co., Ltd. (Shanghai, China). All reagents were of analytical grade and were used directly without further purification.

### 3.2. Preparation of PBMO Heterojunction

Bulk BP was synthesized via a phase transformation technique, as detailed in Reference [63]. A predetermined quantity of I_2_, Sn, and red phosphorus was sealed under vacuum within a quartz tube. The quartz tube was positioned horizontally in a tube furnace (Kejing, Hefei, China) and subjected to heating at a rate of 2 K/min until reaching 923 K, where it was maintained for 5 h. Subsequently, the temperature was decreased at a rate of 0.33 K/min to 773 K and held constant for 2 h. Following this thermal treatment, the sample was allowed to cool naturally to ambient temperature. To purify the product, byproducts were removed through repeated ultrasonic treatment in toluene, followed by refluxing for 15 min, resulting in the isolation of pure black phosphorus crystals.

BP nanosheets were prepared using the following procedure: 0.1 g of bulk BP was dispersed in 20 mL of anhydrous ethanol and subsequently transferred into a 50 mL sealed stainless steel ball milling jar. The milling process employed stainless steel balls of two diameters, 10 mm and 5 mm, with a combined mass of approximately 100 g, evenly divided between the two sizes. The mixture was subjected to ball milling (Nanda, Nanjing, China) at 600 rpm for a duration of 6 h. To remove larger BP particles, the resulting suspension was centrifuged at 5000 rpm, followed by a second centrifugation at 12,000 rpm to isolate the precipitate corresponding to smaller BP particles. Thereafter, 70 mg of the small BP fraction was dispersed in 100 mL of NMP and sonicated in an ice bath maintained at 4 °C for 8 h. This process yielded a brown solution containing BP nanosheets.

The BP/Bi_2_MoO_6_ heterojunction was synthesized using the following procedure: 2 mmol of Bi(NO_3_)_3_•5H_2_O, 1 mmol of Na_2_MoO_4_•2H_2_O, and 0.05 g of CTAB were dissolved in 80 mL of ultrapure water. The resulting solution was stirred for 30 min to ensure complete dissolution, then transferred to a hydrothermal reactor and maintained at 100 °C for 24 h. Upon completion of the reaction, the precipitate was collected, washed five times with ultrapure water, and dried at 60 °C for 8 h. Subsequently, 100 mg of Bi_2_MoO_6_ was introduced into BP NMP solutions of varying volumes (30, 60, 75, 90, and 120 mL), followed by sonication for 3 h and continuous stirring overnight. The mixtures were then centrifuged, washed three times with ethanol, and dried naturally in the dark for 12 h. The resulting samples were designated as PBMO-30, PBMO-60, PBMO-75, PBMO-90, and PBMO-120, corresponding to the respective volumes of BP NMP solution used.

### 3.3. Characterization

XRD patterns were acquired utilizing a Rigaku Smartlab diffractometer (Rigaku Corporation, Tokyo, Japan) with Cu Kα radiation (λ = 1.5406 Å) at operating conditions of 40 kV and 40 mA to investigate the phase structures of the samples. Morphological and structural analyses were performed through TEM and HRTEM using a JEOL-2100F microscope (JEOL, Tokyo, Japan) operated at an acceleration voltage of 200 kV. SEM and elemental mapping were conducted with a JSM-4800F scanning electron microscope (JEOL, Tokyo, Japan). Chemical state characterization was carried out by XPS employing a Thermo ESCALAB 250XI spectrometer (ThermoFisher, Waltham, MA, USA) with monochromatic Mg Kα radiation (hν = 1486.6 eV) at a power of 150 W; binding energy calibration was referenced to the C 1s peak at 284.8 eV. ESR spectra of radicals spin-trapped by 5,5-dimethyl-1-pyrroline N-oxide (DMPO) and 2,2,6,6-tetramethylpiperidine-1-oxyl (TEMPO) were recorded on a Bruker E500 spectrometer (Bruker, Karlsruhe, Germany) under visible light irradiation. Optical properties were examined via PL and time-resolved PL spectroscopy using an Edinburgh FS5 fluorescence spectrometer (Edinburgh, Livingston, UK) at ambient temperature. EIS and transient photocurrent measurements were performed on a Chenhua CHI660D electrochemical workstation (Chenhua, Shanghai, China), employing a 300 W xenon lamp as the visible light source. The working electrode was prepared by spin-coating the catalyst onto a cleaned indium tin oxide (ITO) glass substrate (1 × 1 cm^2^). Light intensity calibration was achieved using a standard silicon photodiode. UV–Vis DRS and UV–Vis absorption spectra were recorded at room temperature with a Shimadzu UV-2450 spectrophotometer (Shimadzu Corporation, Nagoya, Aichi, Japan) equipped with an integrating sphere, using BaSO_4_ as the reference standard.

### 3.4. Photo-Fenton Reaction

The photo-Fenton catalytic activities of BMO, BP, and PBMO heterojunctions were evaluated using an aqueous solution of OTC at a concentration of 20.0 mg L^−1^. In a typical photo-Fenton degradation experiment, 200 mL of the OTC solution was placed in a 400 mL beaker, and the initial pH was adjusted using 0.1 M NaOH or HCl. Subsequently, 100 mg of the catalyst was introduced into the antibiotic solution, which was continuously stirred in the dark at 25 °C for 1 h to establish adsorption–desorption equilibrium. Following this, H_2_O_2_ was added, and the photo-Fenton reaction was immediately initiated under visible light irradiation provided by a 40 W white LED source. At predetermined time intervals, 5.0 mL aliquots were withdrawn and quenched with an equal volume of methanol. The samples were then filtered through a 0.22 μm PTFE membrane to remove the catalyst particles. The concentration of OTC was determined using UV–Visible spectrophotometry.

To assess the effects of light, H_2_O_2_, and catalysts on the OTC degradation efficiency, control experiments were conducted under standardized conditions: initial pH of 7.0, OTC concentration of 20.0 mg L^−1^, H_2_O_2_ concentration of 50.0 mM, and catalyst dosage of 0.5 g L^−1^. The stability of the PBMO-75 heterojunction in the photo-Fenton reaction was evaluated through four consecutive degradation cycles of OTC. After each cycle, the PBMO-75 catalyst was recovered by centrifugation, washed sequentially with ethanol and deionized water to remove the adsorbed OTC, freeze-dried, and then reused in subsequent experiments.

The reactive species trapping experiments were conducted similarly to the photo-Fenton degradation tests, with the exception that 1 mM of specific scavengers was added to the reaction system. Ammonium oxalate (AO), isopropanol (IPA), N_2_, and NaNO_3_ were employed as scavengers to selectively quench holes (h^+^), hydroxyl radicals (•OH), superoxide radicals (•O_2_^−^), and electrons (e^−^), respectively.

## 4. Conclusions

In summary, a novel 0D/2D BP/BMO heterojunction was successfully synthesized via a hydrothermal approach. The combined effects of H_2_O_2_ and visible light irradiation enabled the PBMO-75 sample to exhibit the highest catalytic efficiency, achieving a 92.9% degradation rate of OTC within 40 min. Notably, the catalytic activity of PBMO-75 surpassed that of BP and BMO by factors of 20 and 8, respectively. The formation of the PBMO heterojunction significantly enhanced light absorption across the 200–800 nm wavelength range relative to BMO alone. The superior photo-Fenton catalytic performance of PBMO is attributed to the internal electric field and Z-scheme charge transfer mechanism, which collectively improved the separation efficiency of the photogenerated charge. This improvement facilitated an increased generation of e^−^, •OH, h^+^, and •O_2_^−^, thereby optimizing the utilization of H_2_O_2_ under neutral conditions. This work provides a valuable reference for advancing photo-Fenton catalytic activity through the design of 0D/2D Z-scheme BP-based heterojunctions.

## Data Availability

The original data are available from the corresponding author upon reasonable request.

## Supplementary Materials

The following supporting information can be downloaded at https://www.mdpi.com/article/10.3390/ijms26167751/s1. References [64,65,66,67,68] are cited in the Supplementary Materials.
