# Supplementary material for: Enhanced Photo-Fenton Removal of Oxytetracycline Hydrochloride via BP/Bi2MoO6 Z-Scheme Heterojunction Photocatalyst
Source: Int J Mol Sci. 2025 Aug 11;26(16):7751. doi: 10.3390/ijms26167751 (PMC12386328; doi:10.3390/ijms26167751)
Supplement: Supplementary file 1 [file ijms-26-07751-s001.zip › ijms-3775610-supplementary.pdf]

Enhanced photo-Fenton removal of oxytetracycline hydrochloride via BP/Bi_2MoO_6 Z-scheme heterojunction photocatalyst

Jian Feng[1], Xiaohui Li[1], Xia Ran, Li Wang, Bo Xiao, Rong Li* and Guangwei Feng*

School of Basic Medical Sciences/School of Medical Humanities, Guizhou Medical University, Guiyang 550025, China

* Correspondence: lirong1@gmc.edu.cn (R.L.); fengguangwei@gmc.edu.cn (G.F.)

[1] These authors contributed equally to this work.

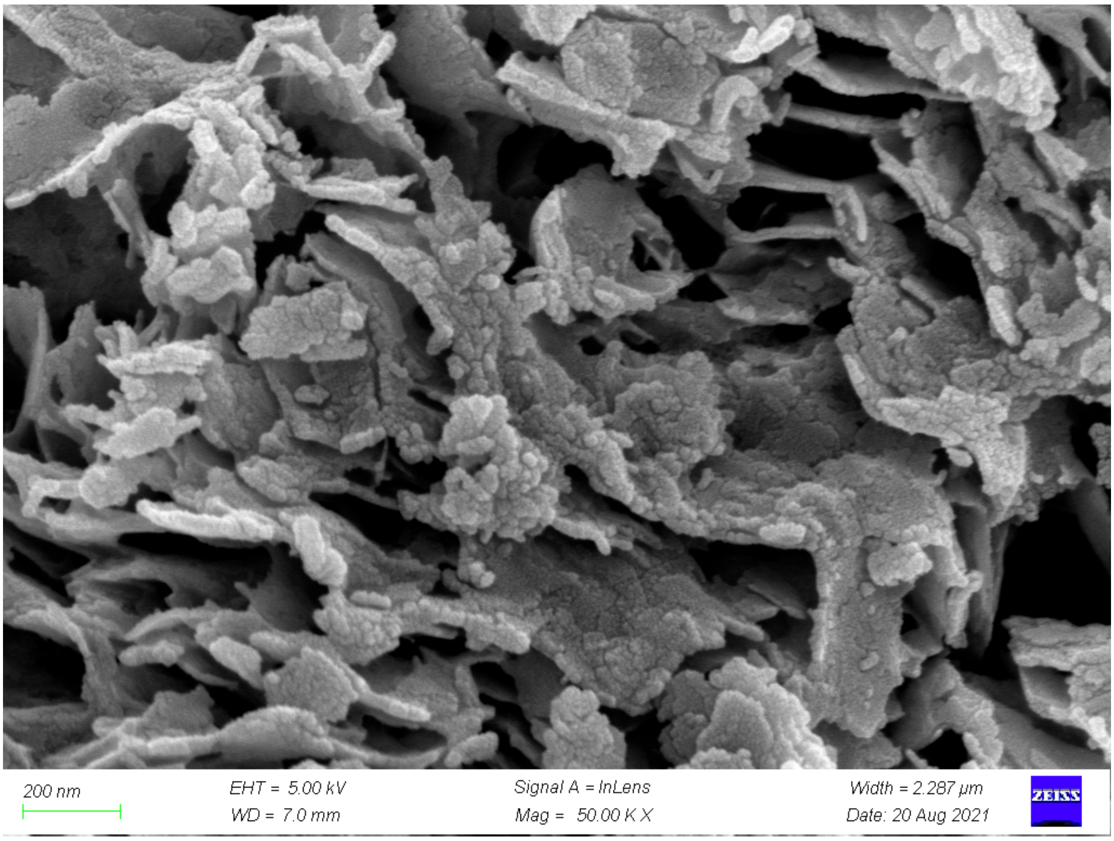

Fig. S1 SEM image of PBMO-75 heterojunction

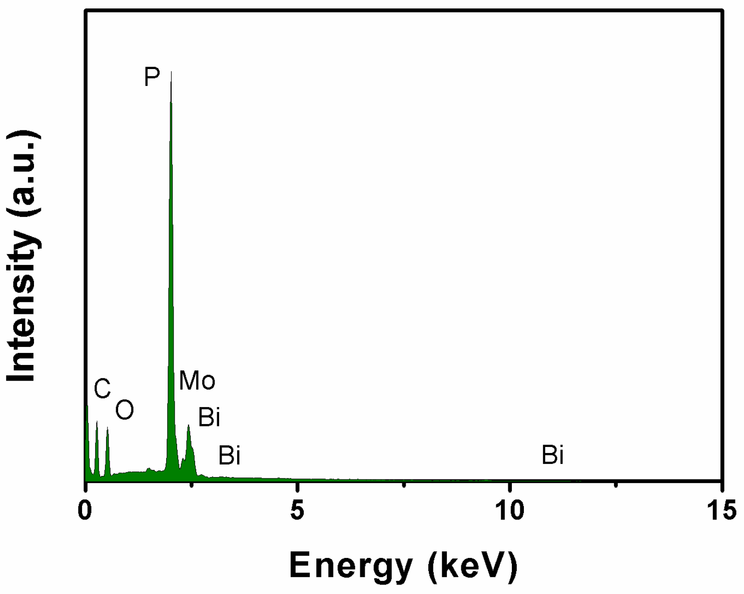

Fig. S2 EDS spectrum of PBMO-75 heterojunction

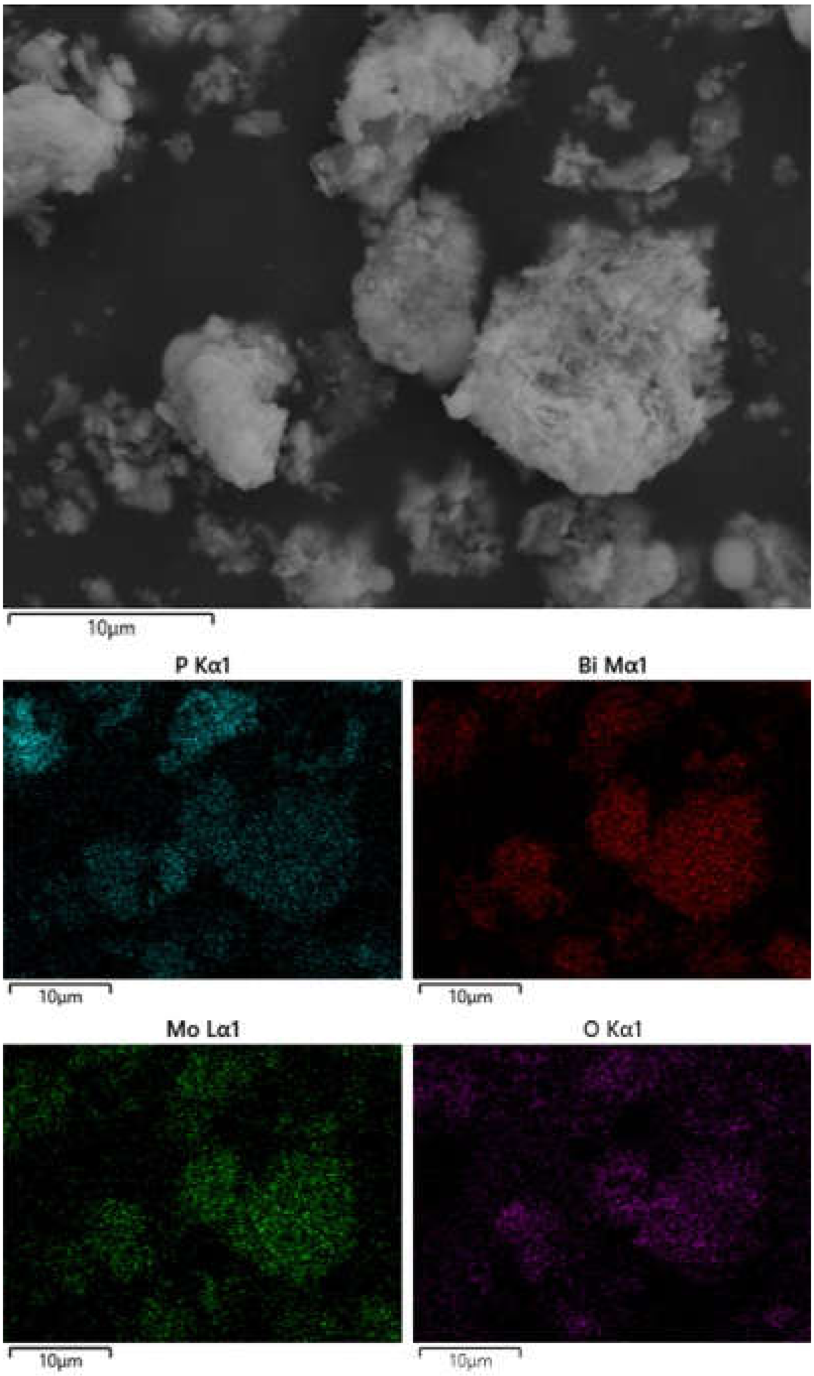

Fig. S3 SEM image and element mapping images of P, Bi, Mo and O of PBMO-75 heterojunction (scale bar=10 μm)

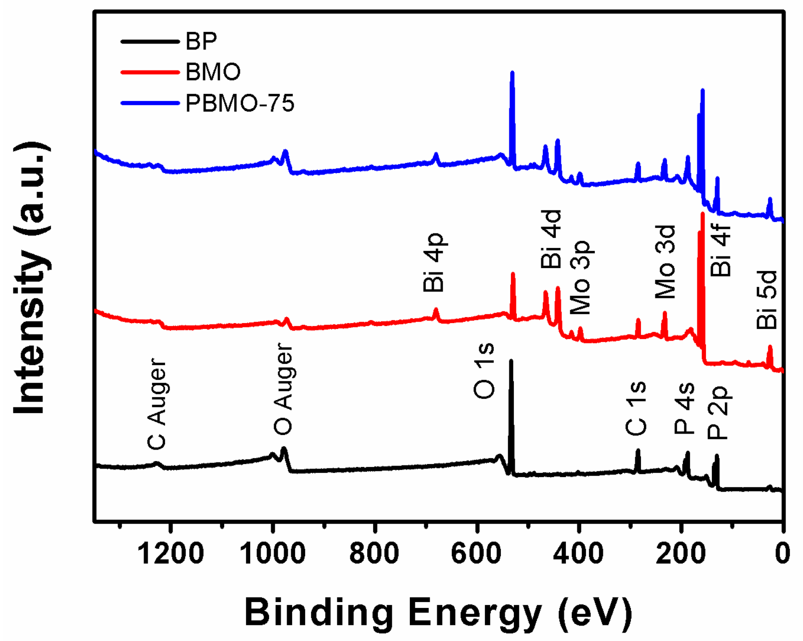

Fig. S4 XPS survey spectra of BP, BMO, and PBMO-75 heterojunction

Table S1. Comparison of photo-Fenton degradation efficiency of OTC on PBMO-75 with various catalysts

Catalysts	Reaction Conditions	Degradation %	Reference
BP/Bi_2MoO_6	20 mg/L, 40 min, 40 W white LED	92.9	this work
$MnFe_2O_4$/g-C_3N_4	30 mg/L, 10 min, 300 W Xe lamp	80.5	[64]
re-Mn-CeO_2	30 µM, 10 min, 500 W Xe lamp	88.1	[65]
Fe_3O_4-Cs	48 mg/L, 120 min, Unknown	over 90	[66]
γ-Fe_2O_3/g-C_3N_4	20 mg/L, 60 min, 300 W Xe lamp	85.7	[67]
Mixed-valent Fe-MOF	20 mg/L, 30 min, 300 W LED	92.6	[68]

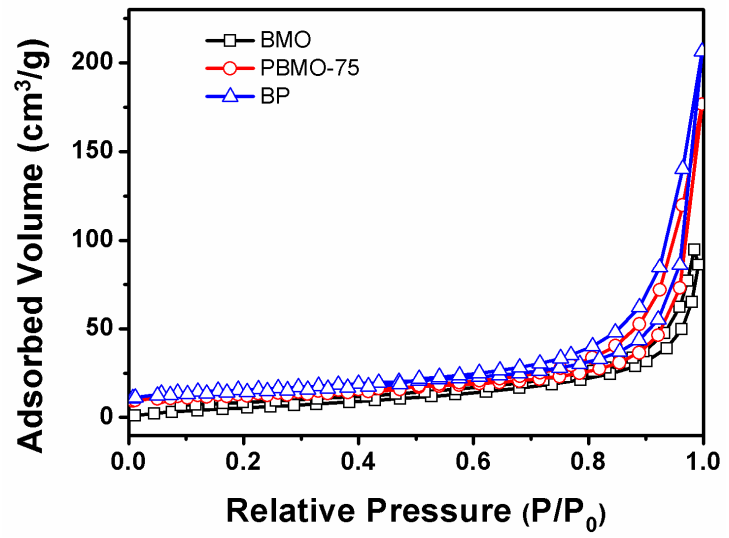

Fig. S5 N_2 adsorption desorption isotherms of BP, BMO, and PBMO-75

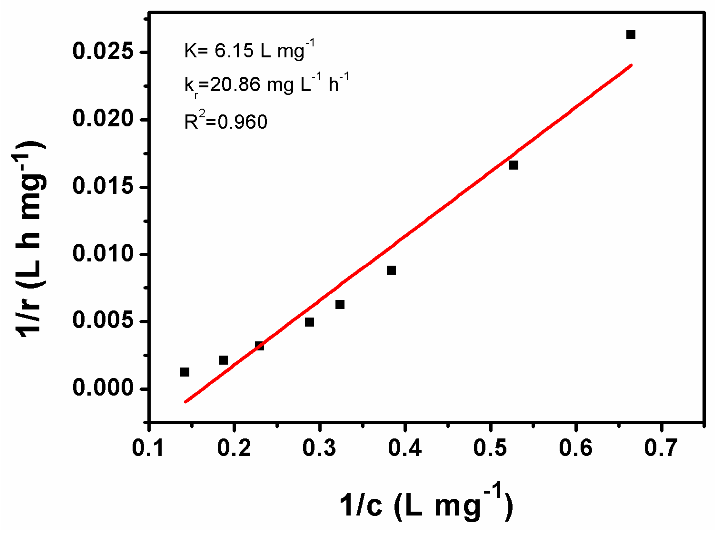

Fig. S6 The degradation kinetics of OTC over PBMO-75 fitted by Langmuir–Hinshelwood model

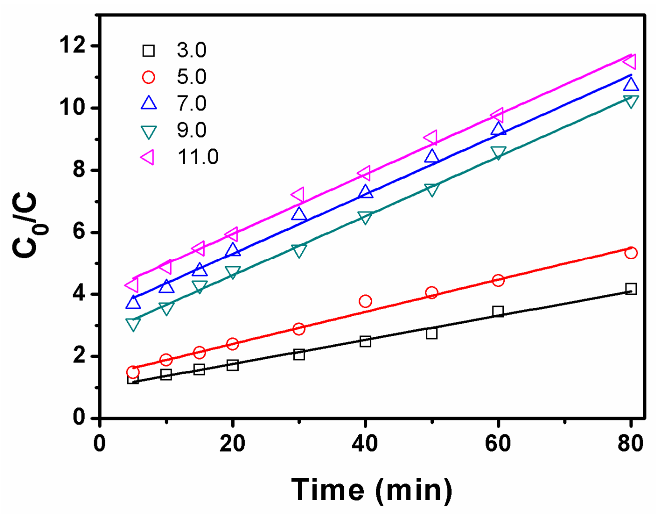

Fig. S7 Degradation rate constants of OTC on PBMO-75 heterojunction at different pH

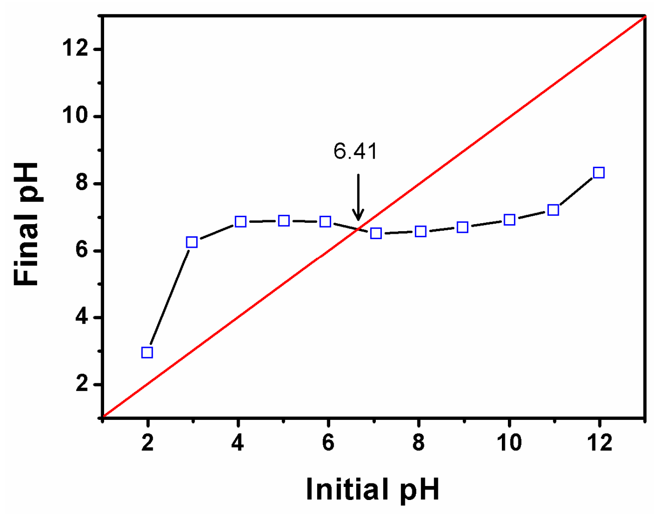

Fig. S8 The point of zero charge (pHpzc) of PBMO-75: Bisector method

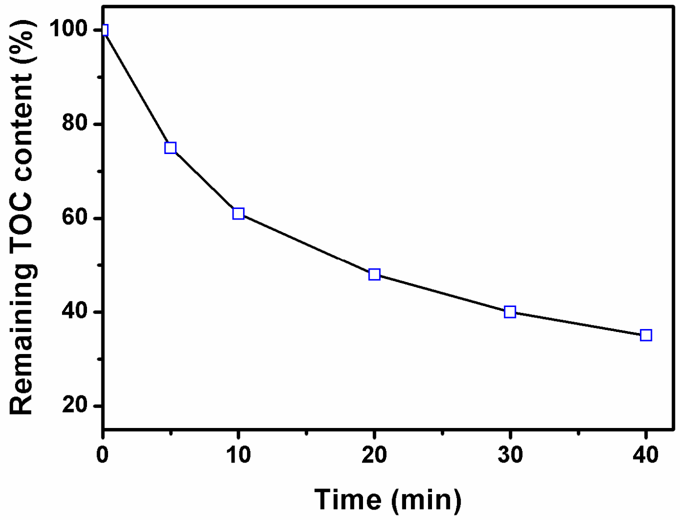

Fig. S9 TOC analyses of OTC photo-Fenton degradation over the PBMO-75 heterojunction

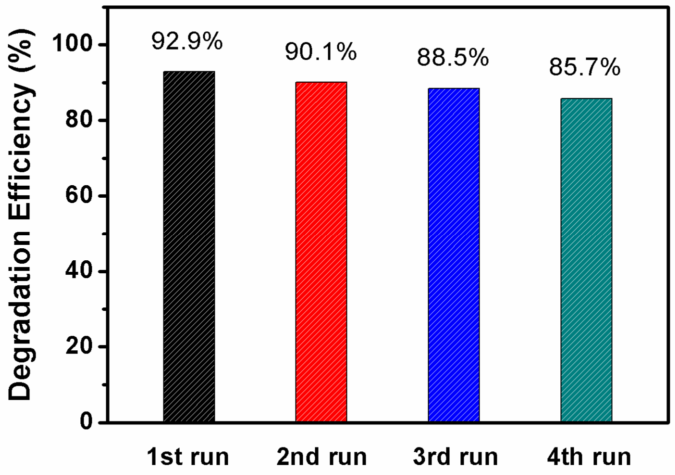

Fig. S10 Performance of PBMO-75 heterojunction in photo-Fenton degradation over four cycles

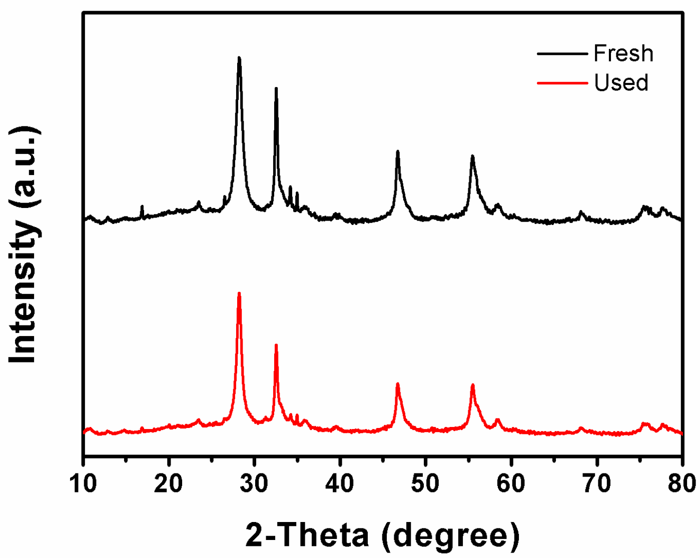

Fig. S11 XRD patterns of fresh and used PBMO-75

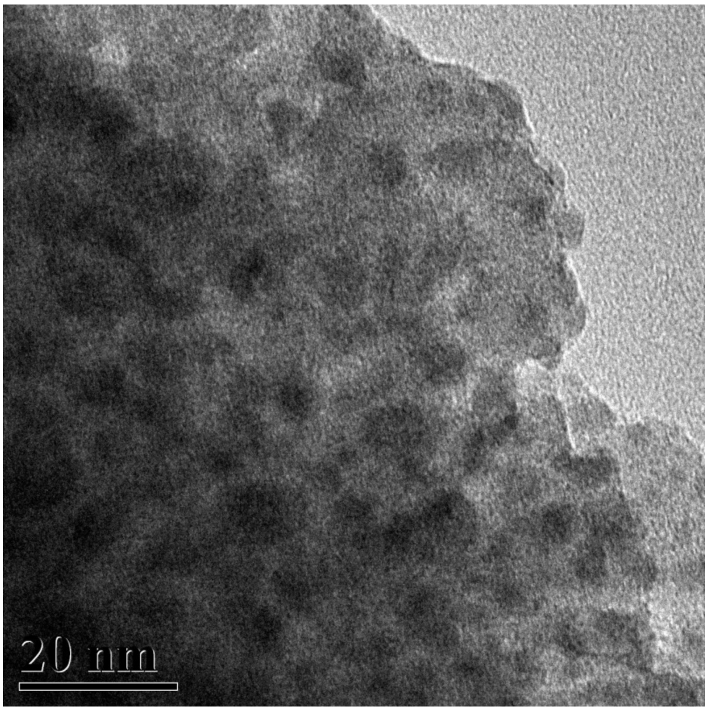

Fig. S12 TEM image of used PBMO-75

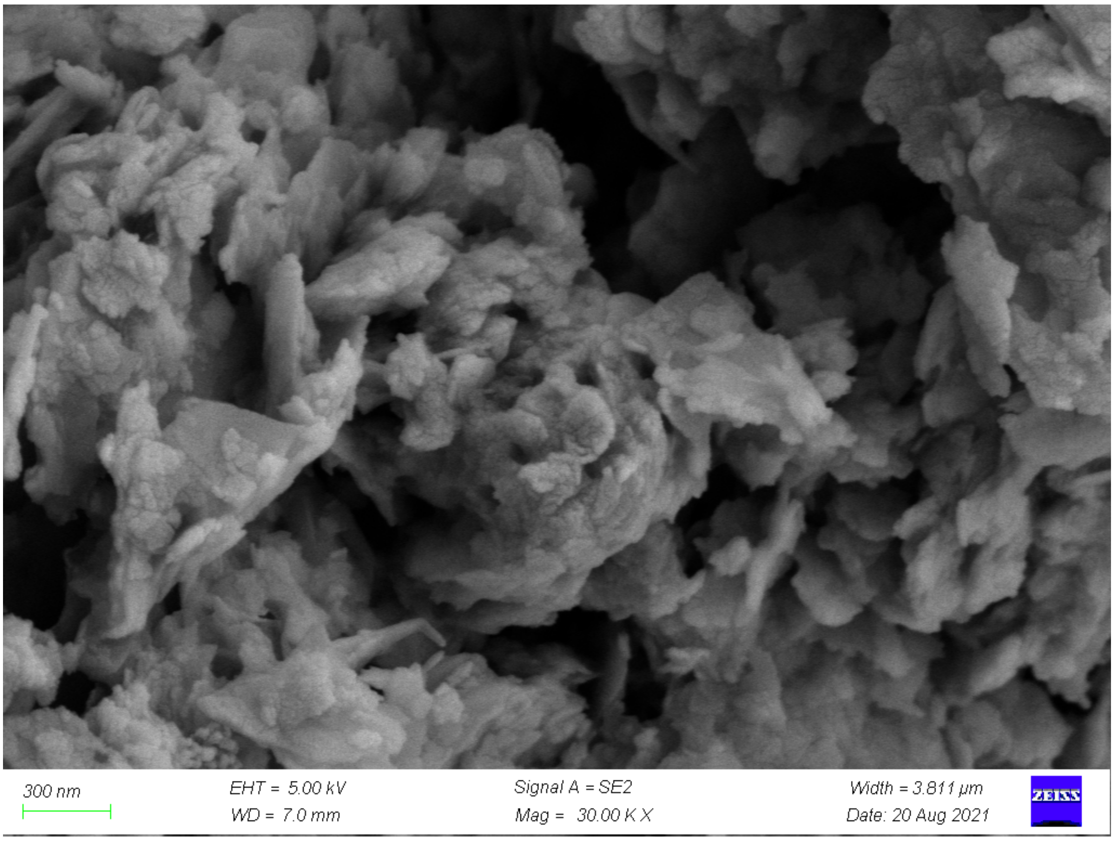

Fig. S13 SEM image of used PBMO-75

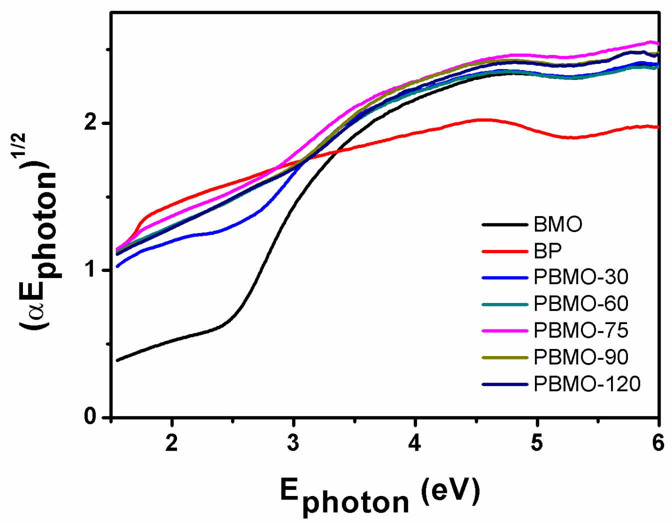

Fig. S14 $(\alpha E_{photon})^{1/2}$ versus E_{photon} curves of the BMO, BP and PBMO heterojunctions
